# Working for patient safety: a qualitative study of women’s help-seeking during acute perinatal events

**DOI:** 10.1186/s12884-017-1401-x

**Published:** 2017-07-17

**Authors:** Nicola Mackintosh, Susanna Rance, Wendy Carter, Jane Sandall

**Affiliations:** 1 SAPPHIRE Group, Department of Health Sciences, University of Leicester, Centre for Medicine, University Road, Leicester, LE1 7RH UK; 2 Institute for Health and Human Development, University of East London, London, UK; 3 Division of Women’s Health, Faculty of Life Sciences and Medicine, King’s College London, London, UK

**Keywords:** Patient safety, User involvement, Maternity care, Obstetric complications, Early warning signs, Help seeking, Obstetric emergency, Safety work

## Abstract

**Background:**

Women and their relatives can play an important role in early detection and help seeking for acute perinatal events. Recent UK reports indicate that patient-professional partnership in ‘working for safety’ can be difficult to achieve in practice, sometimes with catastrophic consequences. This research explored the experiences of women and relatives who had experienced early warning signs about their condition and sought help in escalating care.

**Methods:**

Secondary analysis of case study data which included qualitative interviews with 22 women purposively sampled on account of experiencing a step up in care and 4 of their relatives from two NHS Trusts in England during 2010. Analysis focused on the type of safety work participants engaged in, and the opportunities and challenges reported by women and family members when negotiating safety at home and in hospital.

**Results:**

Women and relatives took on a dual responsibility for self-diagnosis, self-care and seeking triage, whilst trying to avoid overburdening stretched services. Being informed, however, did not necessarily enable engagement from staff and services. The women’s narratives highlighted the work that they engaged in to build a case for clinical attention, the negotiations that took place with health care professionals and the strategies women and partners drew on (such as objective signs and symptoms, use of verbal insistence and repetition) to secure clinical help. For some women, the events left them with a lasting feeling that their concerns had been disregarded. Some described a sense of betrayal and loss of trust in an institution they believed had failed to care for them.

**Conclusion:**

The notion of ‘safety partnerships’ which suggests a sense of equality and reciprocity was not borne out by our data, especially with regards to the experiences of teenage women. To enable women and families to secure a rapid response in clinical emergencies, strategies need to move beyond the provision of patient information about warning signs. Effective partnerships for safety may be supported by system level change such as improved triage, continuity of care, self-referral pathways and staff training to address asymmetries of power that persist within the health system.

## Background

Perinatal emergencies often develop rapidly and unexpectedly and can lead to poor outcomes for mother and/or the baby, making timely escalation of care a high policy and practice priority. Lack of recognition, appropriate treatment and referral of women and babies who have, or are developing, a critical condition during or after pregnancy continue to feature prominently in national and international reports [1–3].

Patients’ and relatives’ tacit knowledge has been found to be useful in early detection of a deteriorating condition [4]. Over the past decade, the importance of this resource, alongside other assets through which patients secure their own safety, has become increasingly recognised. The World Health Organisation (WHO) states that patients should become active partners in improving the safety, quality and efficiency of health service delivery [5]. Patients are increasingly recognised as ‘experts’ in their own illnesses and care, able to usefully participate in recognising and averting errors, near misses and adverse events [6]. This reflects the effects of a wider consumerist movement towards market-orientated models of healthcare which emphasise patient choice, individual responsibility, shared decision- making, partnership and agency [7, 8].

While patients often demonstrate positive attitudes toward promoting their own safety, this does not necessarily translate into actual behaviours [6]. There are also concerns that the shift in role may signify an unwanted burden for some, taking the form of ‘responsibilisation’ rather than ‘empowerment’ [8, 9]. Conceptualisations of involvement in safety typically focus on improving patients’ knowledge at the individual level while neglecting the role that context plays in shaping health [10]. The notion of a ‘safety partnership’ may be problematic given the uneven power dynamics within healthcare and the vulnerabilities of patients and carers [11–13]. Patients have reported feeling uncomfortable about being expected to check up on, or challenge health professionals’ actions, anticipating that this could lead to them being labelled as ‘difficult’ with a negative impact on quality of care received [12]. Patients’ readiness to speak up seems to be affected by the quality and nature of their relationships with staff [14]; patients have been discouraged by having their input ignored or belittled, or by fear of victimisation [6, 15, 16].

What is missing from the current debate is an operational understanding of how local context, patient and family and staff roles and relationships interrelate to affect safety [17]. The notions of ‘expertise’ and ‘partnership’ are mostly used in the context of patients with long-term conditions with regards to their participation in treatment and care management decisions. It is less clear how these concepts apply to patient involvement in safety, particularly in the context of acute episodes of illness. Existing research on patient involvement in safety has tended to be based on hospital or hospice settings, and has typically focused on error prevention (e.g. prompting staff to wash hands and detecting medication errors). Maternity care provides an interesting case study as women navigate care across settings spanning community and hospital care. They also have to negotiate boundaries between their sense of normality and risk. While birth in the UK is generally very safe for the majority of women and their babies, problems in maternity care persist, and these problems disproportionately affect women and babies from some ethnic groups and those who are socio-economically disadvantaged [3]. Recent reports detail examples where women and partners tried to secure help for early warning signs of complications but their concerns were dismissed [2, 18]. This research aims to extend knowledge of the opportunities and challenges associated with patient safety work in escalation of maternity care by situating this type of work, together with patient and provider roles and relationships, within the wider institutional structure of maternity healthcare.

## Methods

This paper is based on a secondary analysis of qualitative data collected as part of a UK- based National Institute of Health Services Research (NIHR) funded programme of work exploring how services are organised to improve patient journeys through and across care systems. Secondary data analysis is considered a useful method of increasing the utility of qualitative data [19]. In this paper we utilise existing qualitative data from two studies, ‘Managing complications in maternity and acute care’ (NIHR King’s PSSQRC) and a Masters in Public Health (MPH) study [20]. Our data set comprised 26 in-depth 60–120 min interviews with 22 women and 4 partners in 2010. The data set included a subset of women and family members’ interviews (*n* = 20) drawn from a wider ethnographic study of the management of acutely ill patients in hospital [21, 22] as well as the complete MPH data set (*n* = 6). Included interviews focused on women’s and family members’ involvement in escalation of care in the management of perinatal complications. Ethical and research governance approval had been obtained for the interviews [08/H0808/178, 09/H0803/143]. Maximum variation sampling [23] had been employed to include women with a range of socio-demographic characteristics who had experienced a step-up in their care, including 6 teenage mothers (see Table 1). The perinatal events ranged from life-threatening episodes to relatively minor complications. Within our sample were two cases of neonatal death (see Table 2).

**Table 1:** Demographics of the interviewees

Age	Women in sample
<20	6
20-29	7
30-39	7
40+	2
Ethnicity	
White (British, Irish, European)	12
Black and minority ethnic (British, Asian, African, Caribbean, Latin American)	10
Parity	
Primiparous	18
Multiparous	4

**Table 2:** Reasons for step up in care

Interviewee (pseudonym)	Reason for step up in care (as defined by midwives caring for the women)
Alice	Transfer in due to meconium stained liquor
Belinda	Transfer into hospital as baby cold on delivery
Carol	Meconium stained liquor, high blood pressure, undiagnosed breach, caesarean section
Daphne	Induction of labour
Edith	Home birth, bleed post partum. 3rd degree tear
Georgia	Induction as late, long labour, ventouse delivery
Helen	Unattended birth
Irene	Baby born quickly on arrival to hospital
Jo	Crash section, Forceps delivery
Kate	Trying for vaginal birth after caesarean section, emergency caesarean section
Lucy	Induction, long labour, ventouse delivery
Mary	Induction, ventouse delivery, episiotomy, bowel and bladder problems
Natasha	Breech, failed attempted to turn baby, needed caesarean section
Olive	Vulval haematoma
Petra	Ruptured uterus
Queenie	Difficult delivery, 3rd degree tear
Rachel	Neonatal death
Sam	Induction, caesarean section
Tracy	Post-partum haemorrhage
Ursula	Midwife unable to hear baby’s heart beat, transfer in
Violet	Failure to progress, caesarean section
Wendy	Transfer in for 3rd degree tear

We use pseudonyms for the interviewees and providers to maintain anonymity. Both sites served a mixed population in terms of ethnicity and socio-demographic characteristics. Each had a maternity service providing care for around 6000 women annually. Eastward had an obstetric unit (mixed high and low risk care environment) while Westward had both an alongside midwifery unit (providing care for women classed as low risk) and an obstetric unit (predominantly high risk environment). Both urban Trusts provided services for home birth and cared for women who were transferred in from home.

For both studies, the interviewers had taken a narrative approach to elicit women’s stories of their pregnancy and childbirth [24]. The women were asked broad opening questions about themselves, about being pregnant and having a baby. While the interviews were semi-structured to explore women’s experiences of deterioration in condition and escalation of care, interviewers took care to maintain the flow of the story. Using a narrative approach allowed the women space for reflection and reconstruction of events from a different point in time. The act of sense-making is itself a construction of a narrative, requiring elements to be selected out, highlighted as significant or surprising, and juxtaposed with one another [25].

All interviews were conducted at the women’s homes apart from one where the woman requested to be interviewed in a coffee shop. Four of the partners participated in the interviews and two volunteered for separate interviews. Interviews were conducted individually by NM, SR (social science researchers, NM with a nursing background), and WC, a midwife. Interviews were recorded and transcribed verbatim. All transcripts were imported into N-Vivo version 8. This facilitated grounded theory and thematic analysis. We focused for the analysis on the type of safety work participants engaged in, notions of safety and what influenced this, and the opportunities and challenges reported by women and family members when negotiating safety at home and in hospital. The following three chronologically ordered themes emerged: becoming informed; feeling safe or unsafe and securing help; and the legacy of acute perinatal events for women and institutions. Table 3 illustrates the subthemes that led to their development.

**Table 3:** Themes and sub-themes

Theme	Sub-theme
Becoming informed	Multiple sources
	Professional encounters
	Being a responsible user
	Cultural, age and language considerations
Feeling safe or unsafe and securing help	Vulnerability due to condition
	Relational trust
	Resources and strategies
	Professional knows best
	Family members role in escalating care
The legacy of acute perinatal events for women and their trust in institutions	Sense making
	Betrayal

## Results

### Becoming informed

#### Multiple sources

We found that women took an active role and engaged in safety work during pregnancy, birth and the postnatal period to keep informed. They were eager to show how they had met their self-care responsibilities by attending health checks by the midwife and reading health information. Their safety work was prompted by a need to understand the risks associated with pregnancy and birth so they could look out for signs of complications.

‘I got in touch with friends who had babies here.[..] I asked [the midwife] some questions when I wasn’t quite sure. I read about everything, online or in books because I wanted to prepare, so I was prepared for the worst, you know, just what can happen’ [Helen]

The women and their partners drew on a variety of informal as well as professionally organised resources. These included pregnancy and childbirth accounts from family members and friends, books, websites and online forums in addition to the literature given out at antenatal visits and discussions they had with providers.

‘I looked at a range of data sources rather than by looking at one single source. Not being a medical professional I’m not really in a position to judge authoritativeness, but I’d look at Wikipedia, at something from a US hospital, something from the UK. I didn’t look into it in great detail but I looked into it in enough detail to feel reassured [Jon, partner of Edith]

A few interviewees reported learning skills from antenatal trainers and also from other women in how to secure access to services in terms of not only when to seek help and who to contact, but what to say and how to behave.

‘I remember the NCT lady saying, ‘When you’re in that waiting room, don’t be polite. If you feel like people are not paying attention to you, don’t allow this to happen. Make a fuss, go back to reception and re-announce your presence.’ [James, partner of Queenie]

#### Professional encounters

Some women noted shortcomings in the way maternity services were organised in terms of meeting their health information needs. They described how antenatal check-ups were organised and delivered to enable the passage of women *through* the maternity system, rather than to promote individualised discussion around their health needs and safety concerns.

‘I love my GP, but he was very quickly nothing to do with the pregnancy. I was put into a midwife system, and the meetings were very perfunctory. It was just, test your urine, test your blood pressure, and fill in the form and book your next appointment for the scan. It felt like it was the bare minimum… of… plotting points’ (Georgia)

‘I do think that if I hadn’t been the kind of person who looks things up… I wouldn’t have been nearly as well-informed… there were lots of things that I discovered from the internet or books that I didn’t find out from the midwives that I saw. The antenatal visits were… quite short and brief, and business like’ (Edith)

Many of the women reported that discussions tended to be orientated around plotting points and verifying progress in the antenatal period. Potential complications that *could* arise later in the perinatal period were not always talked through.

Researcher: Did the midwives check with you that you knew about certain things?

Interviewee: [Hesitantly] N-no. No. Um… I’m trying to think of an example, but… Um… I was generally pretty healthy, and… I had all, I had the symptoms of pregnancy [Edith]

Petra, in contrast, had had a previous caesarean and wanted to try for a vaginal delivery. She was given antenatal advice about early presentation to the hospital to ensure staff could monitor her progress and detect early signs of scar rupture.

‘This is what my midwife was telling me *every* antenatal … clinic, “the moment you go into labour, you have to come to the hospital”, you need to be monitored, you need to see them … if we see the scar is opening we have to rush you to … theatre, we have to remove the baby, and for yourself as well.’

#### Being a responsible patient

Most of the women’s narratives showed how they situated their own individual health information needs within wider system expectations of maternity care. Their experiences were linked to their expectations of maternity service provision within the publicly funded NHS, and perceptions of entitlement and fairness.

‘With all my questions I felt a bit bad because I saw that there were more people waiting, and seeing people waiting outside and knowing that the midwives were only there two days a week, I felt bad having this list of questions to ask and taking up more time.’ [Wendy]

Wendy’s account highlights the social nature of safety work and the trade-offs some women felt obliged to make in balancing their need for information versus others’ requirements given the limited availability of midwives at the antenatal clinic and their perceptions of busyness in midwifery workload. Extending women’s responsibility for safety *beyond* guidance provided at the clinical encounter was considered reasonable, on account of their own abilities and what could be expected from maternity service provision, as Georgia explains below:

‘I’d pretty much seen the same midwife, but she had no recollection of me. There wasn’t any… sense of… oh this is my midwife who’s seeing me through. But I didn’t expect that, because you don’t get that on the NHS. And I didn’t really need it. As long as my questions can be answered and someone’s responding, and making sure there’s no oversight of medical difficulties, I didn’t need to be hand-held’.

#### Cultural, age and language considerations

Some of the women’s narratives demonstrate a level of confidence in articulating their health information needs. Several of the teenagers interviewed described feeling the information they received was not targeted for their needs. The antenatal classes and health education lacked meaning which they found disempowering when trying to use this information to engage with and negotiate access to services.

‘I had antenatal lessons but they didn’t really help. The way they talk about the pain to what it was, was really different. I think that in young people’s cases they should have special lessons, like a course or something that when you are going to give birth they explain more in details to young people ‘cause we don’t know. Some of us don’t even know how we got pregnant’. [Jo]

Some of the women had limited knowledge of English which presented challenges in terms of accessing and sharing information with health staff. The presence of a relative helped overcome some of these barriers. An interpreter helped the start of the relationship between Carol and her midwife, particularly assisting Carol to answer questions about her health status and previous medical history.

‘I wasn’t here, first day, so for my wife it was bit tough that day’ [Steve, partner of Carol]

‘All night long before I don’t sleep. I don’t know who is coming, not my language, will I be handed questions I don’t know? [But this midwife B called this translator]. With translator on the phone, she is very good, all very quick, a lot question I understand, like about me, am I sick, about heart, about family’ [Carol].

### Feeling safe or unsafe and securing help

Having access to health information did not necessarily guarantee the women meaningful engagement with staff and services. Women’s levels and types of participation in help-seeking varied widely, as did the situations that led them to feel safe or unsafe. Their conceptualisations of safety were relational, constantly developed and negotiated with significant others (e.g. partners, relatives, friends, extended family) as well as health professionals. Their ability to contribute to safety was influenced by factors including the nature and trajectory of the acute perinatal event, presence and involvement of friends and family, the nature of relationships with staff, the availability of resources, the setting in which negotiations took place, as well as local social rules and policies.

#### Vulnerability due to condition

Evident in a number of women’s accounts was the rapid onset and presentation of obstetric complications such as post-partum haemorrhage, which made it difficult for them to contribute to early detection and management.

‘In my mind I was like “oh my God I am going to die what is wrong with me”, there were so many people in theatre cause I heard them bringing in some machines. I lost so much blood [..] One of the midwives started crying, I didn’t mind cause I couldn’t cry, she was doing it for me’. [Olive]

*‘*I was really exhausted but had just given birth, I put this down to the fact that I’d pushed hard for a long time. I was feeling fairly out of it but nobody seemed alarmed by it. I remember saying, ‘Oh I feel like I’m in the room but not in the room,’ but… I’ve never given birth before so I didn’t know that that was unusual*’* [Tracy]

By the time that many of the women started to feel signs of physiological compromise, the underlying problem was well advanced. The nature of debilitating and unfamiliar symptoms limited the women’s ability to engage in the most basic of safety acts: mobilising help for themselves and their babies.

#### Relational trust

The quality of patient-professional relationships influenced women’s confidence and agency in contributing to safety, with trust between women, partners and professionals playing a crucial role. Feelings of safety were enabled when women perceived they had had the opportunity to develop a connection with staff and these staff demonstrated presence during periods of vulnerability.

‘I didn’t have any time hardly where the midwife wasn’t with me. I’d got in the ambulance, by the time she’d got in the lift it was literally five minutes, when we did get [to the hospital] I was very dependent on [midwife, C], I found that, because I knew her as well, she kept me so calm and very focused’ [Alice]

Safety was perceived by the women to encompass physical and psychological elements. Professional qualities such as technical expertise in checking for complications and interpersonal skills such as showing compassion and kindness, impacted on individual’s feelings of safety.

‘The midwife was really good. She used to check me all the time, if the baby was OK, if I was OK. She was really good and friendly to me, all my family liked her, she helped me a lot. If I get pregnant again I’ll call her. She was there for me’ [Belinda]

For Tracy and Lucy relational trust was aided by an added element of continuity of care and a relationship built up over time.

‘I went into [my birth] experience with total trust and confidence in my midwife. I don’t say you couldn’t have built that up with someone that you’d just met, but because she was part of the caseload team I went with everything that she suggested. The fact that I felt part of this trusted community set-up gave me extra trust and confidence, and also meant that during the complications, I felt less scared because she was there’ [Tracy]

‘I passed out and woke up sitting up with tubes and stuff sticking out of my hand. [..]The midwives made sure I knew what was going on, they made sure I was comfortable and they listened to me as well, that was important and the fact that they were there the whole time’ [Lucy]

Safety work encompassed aspects of emotional and psychological reassurance as well as physical safety. Women described feeling safe on account of relational trust despite the inevitability of unequal relationships associated with managing acute deterioration.

#### Resources and strategies

A number of women reported that they were in a position to help secure professional response for admission into hospital or escalation of care once already in the hospital setting. Their narratives highlighted the work that they engaged in to build a case for clinical attention, the negotiations that took place with health care professionals and the strategies women and partners drew on (such as objective signs and symptoms, use of verbal insistence and repetition) to secure clinical help.

Petra had been schooled in what to say and what to expect from the hospital staff in response to her going into labour. This authoritative knowledge gave her the confidence to insist on timely transfer to the hospital via the ambulance services.

‘The ambulance people were here, ‘What’s the problem?’ I say, ‘I’m overdue, I’ve seen blood, and I have a scar which needs monitoring. So I need to go to the hospital.’ They said, ‘We can’t take you to the hospital, call the hospital while we are still here, talk to them, let us hear what they are saying.’ I called the hospital and the midwife said, ‘don’t worry, put some water in the bath tub and make a cup of tea and sit in there.’ I said, ‘I was told that the moment I go into labour I should come to the hospital, I need to be monitored.’ The midwife told me, ‘OK, let the ambulance people bring you over to the hospital’.

Safety work was a dynamic process, extended over time and contingent on local context. In Petra’s account, her claims for attention lost urgency as she progressed through the busy maternity system and came into contact with others’ competing requests for triage and clinical response.

‘The ambulance people gave my notes to the [labour ward] receptionist. I waited. I’m in pain. After some time I was like, God, it’s another hour nobody has come to see me, what’s happening? I talked to the receptionist. I told her, ‘Look, I was brought here by ambulance, I’m in pain, I’m overdue, and I have a scar which needs monitoring.’ She told me, ‘You see those two ladies stood there? They came before you.’ I couldn’t understand what was going on. Those ladies were heavily pregnant but they were not in pain, they were stood with their partners, there I was down on the floor’

Boundary distinctions between settings and rules for access at times increased social distance and power differentials between women and staff.

‘We got to the reception desk and the receptionist said, ‘I need the notes.’ Her attitude was just terrible. Obviously in the rush to get me out, the notes had fallen [out of the bag]. My husband hadn’t noticed. She wouldn’t let us in without the notes’ [Rachel]

While boundaries, standards, protocols and guidelines had been established to structure service provision, improve safety, protect resources for those in most need and maintain efficiency, at times women and relatives struggled to make sense of these professionally defined rules.

#### The professional knows best

The women’s ability to negotiate safety was also significantly influenced by their position as patients within the maternity pathway because of associated norms and codes of conduct. They described trusting hierarchies of professional over embodied knowledge, which meant that at times they downplayed their own interpretation of events in the face of professional insistence of an alternative explanation.

‘[We had been sent home]. The midwife had said it takes at least ten hours because every centimetre takes an hour. And then the pain was getting different, I was bleeding like slimy blood, and my partner called the hospital and they were like, ‘Oh that’s still OK, she doesn’t need to come in yet’. He didn’t challenge this because we trusted them and it wasn’t even… near ten hours’ [Helen].

Relying on professionals who they perceived as more knowledgeable, authoritative and culturally able to secure their safety, at times created a sense of cognitive dissonance for women and their partners. When the safety actions taken by professional staff did not align with what they had read or believed to be most appropriate action, these women described feeling anxious and unsafe. Yet these feelings did not necessarily equip them to raise their concerns with staff.

‘My water colour change. Then I call. And the midwife said, ‘what colour is it?’ I say, ‘It’s green’ She said ‘Wait for me, I’m coming nine-thirty’. This for us strange because what I read, when you have this colour you must straightaway go to hospital. And… and the midwife says, ‘No, wait, like four, after four hours I am coming.’ And we scared, oh my God, what? Four hours? This is too long’ [Carol]

Some of the teenage women reported being aware of power imbalances associated with age and parity differences and their lack of experience of navigating the maternity system.

‘I had never had a baby, I don’t know anything. I was thinking like they have been delivering babies for long time so they know what they are doing’ [Mary]

Jo and Kate, also younger women, described feelings of anger and frustration at the difficulties this perceived power imbalance presented for them in terms of actively contributing to their safety. Negotiating for safety was orientated within wider social networks and linked to women’s sense of self.

*‘*I knew most of my waters went … they just said “no your waters is fine”. I was saying “yes some my waters have gone, I told you” what could I do? They didn’t believe me, I had no choice*’* [Kate]

For Jo, moral and emotional dimensions were also embedded within relations between her, her family members and staff.

‘After my birth a midwife said they should move me back into the hot room and I’m like ‘I am not moving because I was numb from the neck all the way down and my baby was crying and he was hot’, and then we got into a big argument and then she said “what do I know about kids after all I am young” so I just felt really judged … when.. my foster carer wasn’t there and my sister was gone’ [Jo]

In contrast, Jon and Alice reported feeling able to counter the decisions made by professionals in the postnatal period regarding the safety of their newly born babies. A complex interplay of factors such as their previous relationships with staff, perceived self-efficacy in caring for the baby and trust in their own judgements enabled them to negotiate on behalf of their children.

‘We felt that the same person didn’t see the baby, Patrick, twice; and for completely understandable reasons they didn’t come when we were told they would. After 72 hours we were told that we might have to stay a bit longer. We felt that Patrick was actually going to be fine at home, and we would know enough to ring up the hospital ourselves. I remember indicating very strongly that… we were going to go home and the doctor was… fine about it in the end’ [Jon, partner of Edith]

Alice discounted the professional telephone advice she received about the significance of her baby’s symptoms, when her daughter’s condition deteriorated soon after discharge home. After a short delay, Alice took the baby to the local emergency department where she was diagnosed with pneumonia and admitted to a high dependency unit.

‘Being a first-time mum I knew there was something not right but I didn’t want to be one of them ones to run straight up to A&E. So I rang NHS Direct and a nurse rung me back, I put the phone up to the baby, you could hear her rattling, she was gasping for breath really badly. The nurse said, ‘It just sounds like she’s got the snuffles.’ I said, ‘But it’s not.’ The GP said, ‘She’s got the snuffles, there’s nothing wrong with her’. It was no help to me whatsoever, so I carried on keeping her cool … it got to half six in the morning and we said, ‘Look, something’s not right, we need to go to the hospital’

#### Family members’ role in escalating care

Presence or absence of family and friends played a part in enabling or negatively impacting on women’s perceptions of safety. A number of women described feelings of vulnerability and disempowerment on account of being unaccompanied, as Kate explains.

‘There was not enough [staff] to help you, they left you for hours. You could press the buzzer and no one would come… I was breaking down and needing help and no one was there to support me or help me. My husband was home with our other son’ [Kate]

Safety work encompassed roles taken on by family members and friends. The women’s accounts showed that those who accompanied them helped secure professional response and contributed to women’s emotional and physical safety.

‘I kept on asking the midwives [about the vulval swelling] and they were like “it is fine, it is fine.” [..] ‘My mum was getting quite worried, “this is not right I have never seen this”. The doctors were like “yeah, it is normal.” And then one of my Aunties came, she looked at it and said “Oh my god your uterus is going to explode.” I was so scared. At that time they weren’t giving me antibiotics or tablets for the swelling. It was only after my Auntie and my mom complained to them that they started to give me antibiotics and everything’ [Olive]

Family members provided a safety net as exemplified by Olive’s account and were able to take on an extended advocacy role.

### The legacy of acute perinatal events for women and their trust in institutions

#### Sense making

Part of women’s recovery process after experiencing an obstetric complication was coming to terms with the seriousness of the event, the way it was managed and the outcomes that resulted. In sharing their narratives, women made sense of events and responsibilities post hoc. Women’s awareness of the outcomes helped shape their perceptions.

‘At the time you’re so grateful to get out of it alive that you don’t think, why did nobody pick up for two hours that I was having an internal haemorrhage? Should they have picked up my blood pressure perhaps a bit more? A bit earlier? It ended up that I ended up losing three litres of blood so that’s seven pints of blood’ [Tracy]

‘It’s hard to say, because I’m not in the medical profession myself, I don’t know whether there was more time they could have given my baby in hospital. Or maybe she could have got a bit more of a health check when she came out of hospital. Or maybe when the midwife come round, check her out a little bit more thoroughly. I’m just thinking… if I knew that there was, you know, there was something wrong with the child I was quite surprised how no one else, didn’t pick up on it, you know? [Alice]

Dealing with the trauma of the events involved the women making sense of the collective nature of patient safety and the precarious nature of childbirth with its inherent clinical uncertainties. The women reflected on staff’s ability to detect and manage deterioration; for some women, this process unsettled their trust in professional expertise and institutional reliability.

#### A sense of betrayal

A number of women sought to minimise the importance of their feelings of betrayal by staff on account of the outcome of their ‘healthy baby’. After requesting not to ‘be cut’, Mary was left with severe bowel problems after an epidural and ventouse delivery. Despite emotional and physical problems she was quick to highlight that her health was improving and her baby was well.

‘The midwife said to me [the ventouse delivery] would help me push cause I couldn’t push […] But, afterwards I was like why did you say “yes” cause the cut was really bad and I wasn’t happy with it. My back passage.. if I have to open my bowels, I have to stay there for two hours waiting and they say “you can not push”. But it is fine. My baby is fine and I am fine’ [Mary]

Two women who experienced the loss of a baby explained the force and legitimacy of their feelings of betrayal and break-down of trust in the institutions they believed had failed to care for them with reference to the catastrophic impact of these events. Petra and Rachel reported psychological and emotional as well as physical harm linked to staff failure to listen to their safety concerns. Feelings of betrayal were linked to a perceived lack of staff compassion, empathy, care and trust at a time of vulnerability for the women when they had no choice but to rely on staff to provide safe care.

‘I was just so embarrassed that I was so wet, my shoes were squelching, I was making so much noise, and they were watching me. Yet this is a caring profession, they should have jumped up and helped me’ [Rachel]

Both women described acting to protect their own safety and trying to raise the alarm but reported being dismissed in their capacity as knowers because they had less medical knowledge, were less familiar with the health system, and did not have access to the frames of reference that the clinicians they come into contact with were drawing on for their decision-making.

‘Didn’t I look like someone who is in pain? I don’t know. Did the receptionist want some money from me? I don’t know. I just ask questions and I have to answer them … by myself… Was I supposed to tell the ambulance, ‘Look, don’t leave me here, take me to another hospital’? I don’t think so. I don’t know’ [Petra]

‘[The midwife] should have picked up the urgency at home, that things were moving quickly, and even if she didn’t believe me she should have worked with me, she should have listened to me, and she should have placated my fears by checking me when I got in there, by examining me when I was asking her to. She’s… working with me to deliver a baby, we’re supposed to be a team, she’s not supposed to be telling me what to do and telling me when I’m wrong’ [Rachel]

The women reflected on their encounters and negotiations, and considered their own roles and responsibilities during these as well as those of the staff they entered into a relationship with. The nature of their adverse experiences shaped ongoing relationships with staff and institutions, and subsequent journeys through the NHS.

‘[Now] even the people who are qualified do their jobs, I don’t trust them, even if I go to the GP [..] I don’t think he’s doing the right thing on me’ [Petra]

## Discussion

Patient safety has been seen historically as a technical issue, rather than a site of organisational and professional politics [26], with patients themselves largely absent from the discussions [8, 27]. Our research contributes to an emerging international body of work that examines patients’ and families’ capacity to provide vigilance over both the patient’s health condition and the care that is given [9, 16, 28]. Our research adds a lens on women’s contributions to safety within the perinatal period, where adverse events often unfold rapidly and can affect the woman and her unborn or newborn baby. Our findings extend insights into how women conceptualise risk and safety, the complexities of the networks that women come into contact with and are part of, the safety work they engage with and how this is also conditioned by the clinical uncertainties, organizational pressures, resource inadequacies and efforts at professional boundary maintenance that make patient safety so hard to maintain [26]. Our research adds to sociologically informed research on safety which has to date largely focused on staff perspectives and professional work (e.g. [29]).

Patient safety engagement models have directed efforts into patient education, aiming to improve ‘safety literacy’ by giving patients information and the ‘right tools’ [9]. A market for pregnancy mobile apps is emerging, bringing digital technology into interventions promoting engagement and participation [30, 31]. Our data problematise notions of patient information as a transformative tool in itself [32]. Some of the women we interviewed were socially networked and reported access to multiple sources of information, as other studies have found [33]. However, being informed did not necessarily equip women to secure help. There is great variability in the quality, accuracy and authority of information on websites, which may create problems for women seeking guidance on early recognition and help seeking [34].

There are additional complexities around the boundary between physiology and pathology in maternity. Midwives may practice ‘verbal asepsis’ [35, 36], and limit conversations about potential complications and warning signs in an effort to avoid medicalisation of normal pregnancy and birth. The recent Montgomery v Lanarkshire Health Board ruling by the UK Supreme Court confirmed women’s right to make autonomous decisions about pregnancy and birth, and to be given sufficient information about ‘any material risk’ that could influence these decisions [37].

Patients’ often hidden role in safety work has long been recognised in the sociology of medical work [38]. The construct of candidacy also holds relevance for our findings as it draws attention to the continual constituting, defining and negotiating process involved in securing help [39]. Our data highlights the complex nature of safety work women participate in, which includes self-monitoring, self-diagnosing, constructing a plausible case for clinical staff, navigating services and checking up on health professionals and care delivery, during their journeys into and within the hospital setting. Rather than perceiving involvement in safety as normative and tied to particular activities and roles, our data points to the dynamic and contingent state of safety relationships [11].

The women’s interview narratives extend existing insights into professional problems associated with escalation of care linked to hierarchies, boundaries and asymmetries of power [40, 41]. Whereas previous studies have found the hierarchical, elitist and paternalistic culture of the health system can be a barrier to patients’ *willingness to engage* with their safety [12, 42], our research demonstrated that women and family members did participate but with variable effect. Their safety work, like that of professionals, is influenced by the social forces that are an inescapable feature of any organisational system [43].

Asymmetry lies at the heart of the clinical encounter [44]. It is a functional asymmetry, reflecting the wider purpose of the institution of medicine in society [44]. Patients are placed in a double bind by the sick role, as they are expected to use their own judgment in determining when it is appropriate to seek professional advice but then to demonstrate their co-operation with legitimate expertise by deferring to the professional’s judgment [45]. Any response to the professional assessment that challenges this asymmetry undermines the patient’s grounds for seeking professional medical help in the first place [44].

Our data demonstrates the nature of ‘epistemic injustice’ [46] when women are dismissed in their specific capacity as knowledgeable [47]. The division of labour that results from increased specialisation in health systems brings problems of coordination, communication, and cooperation [48]. It is no longer considered possible for one person to hold all the specialist knowledge needed to treat patients with complex needs. Knowledge held by staff is often partial or incomplete [49]. Patients and family members are often in the privileged position of holding critical information yet they can find it difficult to persuade staff who are higher-up in the hierarchy of the organization of the credibility of their knowledge or relevance of their perspectives [50]. Our findings suggest the importance of family members’ advocacy roles and women’s heightened vulnerability when left to negotiate safety on their own, particularly with rapidly deteriorating conditions.

The concept of safety partnerships [51] suggests a sense of equality which was not borne out by our data, especially with regards to the experiences of the teenage women. Variations in stillbirth rates have been linked to system level structural inequality, including racism and differentials in access and opportunity [52]. Stigma and discrimination has been reported in maternity care in high resource countries [53, 54]. The effects of workload and resources on provider motivation are recognised as important contributors to disrespect and abuse in childbirth [55]. We need to develop robust ways to generate greater insight into organisational responsiveness to patients’ contributions to safety. The addition of a question to the annual CQC survey on organisational response to women raising concerns during labour and birth for example, has highlighted that 18% of women felt that their concerns were not taken seriously and responses varied considerably across sites [56].

Our data highlights the complex relationships between trust, power and expertise. There are moral and emotional dimensions to patient safety which encompass judgements about appropriate behaviour for women and staff, and claims to legitimacy within the field. The language of patients ‘speaking up’ and ‘challenging’ potentially frames patient or practitioner behaviour as problematic [57]. A theme related to birth trauma is betrayal of trust and powerlessness [58]. Patients may experience a loss of trust in the competency or integrity of their care providers, if they perceived that they had to question or direct staff to take action to address lapses in their care [12]. In the aftermath of harm, individuals may feel that doctors, healthcare organisations and those responsible for regulating health professionals had let them down [27]. Our research provides rich detail how contributing to safety has implications for ongoing relationships and women’s trust in staff and health systems [59]. It also raises broader issues of justice and accountability when harm occurs, and questions about the power and control of organisational systems [27].

Continuity of care models provide an area worth future exploration. Qualitative research findings indicate that advocacy, trust, choice, control and listening to women are important processes underpinning the improved outcomes and experience associated with relational continuity [60]. Outside maternity, relational models of care have been shown to impact on clinical outcomes; similar benefits have not been found from management continuity or information continuity alone [61]. Continuity models in maternity appear to facilitate the health care professional being oriented towards the woman and her community, rather than towards the needs of the institution [62]. They can also confer benefits either as a direct effect of midwives developing trusting relationships with women, or through additional advocacy and gatekeeping roles [63]. The recent National Maternity Review in England and Scotland has highlighted the importance of continuity from a service-level perspective [64–66].

As our secondary analysis draws on data from contextual studies carried out over 6 years ago, an important consideration is whether these findings hold relevance for current practice. UK initiatives introduced since data collection include use of friends and family test [67], real-time feedback and the safety thermometer [68] which arguably could have improved asymmetries of power. However, recent reports such as Kirkup [2] and MBRACE [18] highlight continuing evidence of poor frontline and organisational response to women and families who speak up about safety, and the catastrophic consequences that can ensue when they are not heard. The recent report of women’s experiences of care, Support Overdue [69], shows a maternity system struggling to provide personalised, responsive care, given that half of all women surveyed reported ‘red flag events’ (indicating dangerously low staffing levels). One in five women were not able to see a midwife as often as they required post-birth. Of these women, a third reported that this resulted in a delay in a health problem for them or their baby being diagnosed [69]. Reed’s study of interpersonal factors influencing women’s experience of trauma highlighted that women continue to report experiences of practitioners’ disregard for their embodied knowledge, including their own assessment of labour progress and fetal wellbeing [70].

## Conclusions

This paper outlines the practice of negotiating for safety, and the spectrum of ways safety work played out to positive and negative effect on women’s experiences. Health systems have traditionally attempted to institute cultural change by addressing one dimension of organisation at a time, e.g. staff attitudes and behaviours or protocols [71] without considering dependencies and interdependencies [50]. Improvement programmes need to involve women’s perspectives in their development to ensure appropriateness and effectiveness [72]. Our data suggests useful areas to explore in maternity include structural strategies (e.g. rights-based approaches) as well as self-referral and triage pathways, continuity of care models, and joint women and staff training programmes [73].

## Abbreviations

A&E: Emergency Department
GP: General Practitioner
NCT: National Childbirth Trust
NHS: National Health Service
UK: United Kingdom
US: United States of America
